# Multi-Level In Situ Surface Modification of Electrospun Tetragonal BaTiO_3_ Nanofibers for High-Performance Flexible Piezoelectric Energy Harvesters

**DOI:** 10.3390/ma19081515

**Published:** 2026-04-09

**Authors:** Zijin Meng, Quanyao Zhu, Qingqing Zhang, Huajun Sun

**Affiliations:** 1 State Key Laboratory of Silicate Materials for Architectures, Wuhan University of Technology, Wuhan 430070, China; 344914@whut.edu.cn (Z.M.); 345339@whut.edu.cn (Q.Z.); 2 School of Materials Science and Engineering, Wuhan University of Technology, Wuhan 430070, China; cglamri@whut.edu.cn; 3 Advanced Ceramics Institute of Zibo New & High–Tech Industrial Development Zone, Zibo 255000, China

**Keywords:** electrospinning, BTO nanofibers, core–shell structure, surface modification, two-step annealing, flexible piezoelectric composites

## Abstract

The practical application of inorganic ferroelectric fillers in flexible piezoelectric composites is critically constrained by low polarization efficiency and severe interfacial incompatibility with polymer matrices. Herein, we report a multi-level in situ surface modification strategy that simultaneously addresses both limitations. High-purity one-dimensional tetragonal barium titanate nanofibers (BTO NFs) are first synthesized via sol–gel electrospinning combined with a two-step gradient annealing process, which precisely controls phase evolution and preserves structural continuity. To overcome the detrimental acid-induced degradation of BTO NFs during functionalization, a polydopamine (PDA) buffer layer is first conformally coated, followed by the liquid-phase deposition of a conductive polypyrrole (PPy) shell, forming a robust core–shell PPy@PBT NFs architecture. Incorporating only 4 wt% of these multifunctional fillers into a poly(vinylidene fluoride) (PVDF) matrix yields a dramatic enhancement in electromechanical performance. The resulting flexible piezoelectric energy harvesters achieve a piezoelectric coefficient (d_33_) of 28.7 pC/N, an output voltage of 13 V, and an output current of 0.7 μA, representing substantial improvements over unmodified filler systems. This synergistic enhancement originates from the PDA-mediated interfacial stress transfer and the PPy-induced Maxwell–Wagner polarization intensification, establishing a robust and generalizable paradigm for high-performance flexible piezoelectric composites in self-powered wearable electronics.

## 1. Introduction

With the development of microelectronic devices towards miniaturization and integration, barium titanate (BaTiO_3_, BTO), as a typical perovskite ferroelectric material, has been widely applied in piezoelectric and dielectric energy storage devices owing to its excellent dielectric and piezoelectric properties [1,2,3]. To integrate the high piezoelectricity of ceramics with the flexibility of polymers like polyvinylidene fluoride (PVDF), researchers have extensively explored the incorporation of various BTO nanostructures into PVDF matrices [4,5,6]. However, the practical integration of BTO into flexible polymer matrices faces two fundamental challenges: (1) low polarization efficiency arising from poor interfacial compatibility, and (2) the inability to maintain high piezoelectric activity under mechanical deformation [7,8].

One-dimensional (1D) BTO nanostructures, particularly nanofibers, offer a compelling solution compared to their zero dimensional counterparts. Their high aspect ratio and geometric anisotropy enable more efficient stress transfer from the soft polymer matrix to the rigid ferroelectric phase, while also facilitating the formation of three-dimensional interconnected networks that enhance overall composite performance [9,10]. Nevertheless, the synthesis of structurally robust, phase-pure 1D BTO nanofibers remains nontrivial.

Among the preparation processes for 1D BTO nanofibers, the hydrothermal method yields high crystallinity but makes it extremely difficult to precisely control the 1D morphology, which is highly prone to agglomeration [11]. Electrospinning technology is frequently combined with the sol–gel method and subsequent high-temperature annealing, this approach has emerged as an ideal choice for fabricating continuous ceramic nanofibers because it perfectly balances between a 1D continuous morphology and a high aspect ratio [12,13,14]. However, a critical bottleneck lies in the post-annealing process, where the removal of organic templates must be carefully balanced against fiber fracture, pore formation, excessive grain growth, and undesirable phase transitions [15,16]. Despite its widespread use, systematic studies on the synergistic evolution of crystallographic phase and fiber morphology during annealing—particularly the interplay between precursor chemistry, heating rate, and temperature regimes—remain conspicuously absent [17,18,19].

Even when high-quality BTO nanofibers are successfully fabricated, their incorporation into polymer matrices introduces additional interfacial challenges. The stark mismatch in elastic modulus between inorganic BTO and polymers such as poly(vinylidene fluoride) (PVDF) leads to stress concentration, interfacial debonding, and inefficient poling [7]. To address this, various surface modification strategies have been explored, including the use of silane coupling agents and the deposition of conductive layers [8,20]. Nevertheless, these approaches often fail to simultaneously provide both chemical protection and interfacial functionality. In particular, the deposition of conductive polymers such as polypyrrole (PPy) typically requires acidic conditions (e.g., FeCl_3_ as an oxidant), which leads to the severe dissolution of the inorganic skeleton, structural collapse, and permanent loss of ferroelectricity [21].

Inspired by bio-adhesion mechanisms, polydopamine (PDA) has emerged as a versatile surface modifier capable of forming conformal coatings. Importantly, PDA serves not only as an adhesion-promoting layer but also as a protective barrier against harsh chemical environments [22,23]. This dual functionality remains largely underexplored in the context of ferroelectric fiber modification [24]. Herein, we propose a multi-level in situ surface modification strategy to construct a robust hierarchical core–shell structure (PPy@PBT NFs), which not only shields the BTO core from acid etching during the subsequent PPy deposition but also mitigates the elastic modulus mismatch between the rigid ceramic and the soft PVDF matrix.

By elucidating the mechanisms of phase evolution, morphological control, and interfacial polarization, we subsequently demonstrate how this synergistic multi-level modification significantly enhances the piezoelectric coefficient d_33_ and electrical output of flexible piezoelectric energy harvesters (PEHs). This work establishes a robust and scalable paradigm for high-performance flexible piezoelectric composites, providing an efficient method for structuring and functionalizing ferroelectric nanomaterials, and helping to address long-standing challenges in the integration of next-generation self-powered electronic materials.

## 2. Materials and Methods

### 2.1. Materials

PVDF power (Solvay 6020, Mw ≈ 800,000) was supplied by Solvay S.A., Brussels, Belgium. N,N-dimethylformamide (DMF, 99.8%), glacial acetic acid (99.5%, polyvinylpyrrolidone (Mw = 1,300,000), dopamine hydrochloride (98%), barium acetate (99.5%), tetrabutyl titanate (99.0%), acetylacetone (99.0%), absolute ethanol (99.5%), Tris-hydrochloride buffer (PH = 8.5), pyrrole (99.5%), iron(III) chloride hexahydrate (99.0%), and ethylene glycol monomethyl ether (99.5%) were obtained from Aladin, Shanghai, China. Acetone (99.5%) was supplied by China National Pharmaceutical Chemical Reagent Co. (Beijing, China).

### 2.2. Preparation of 1D BTO Nanofibers

In this experiment, one-dimensional tetragonal barium titanate nanofibers with a dense structure, smooth surface, and pure crystalline phase were first prepared. The specific preparation process involved three steps: the preparation of barium titanate sol, electrospinning, and two-step annealing.

#### 2.2.1. Sol-Gel Preparation

**Figure 1:**
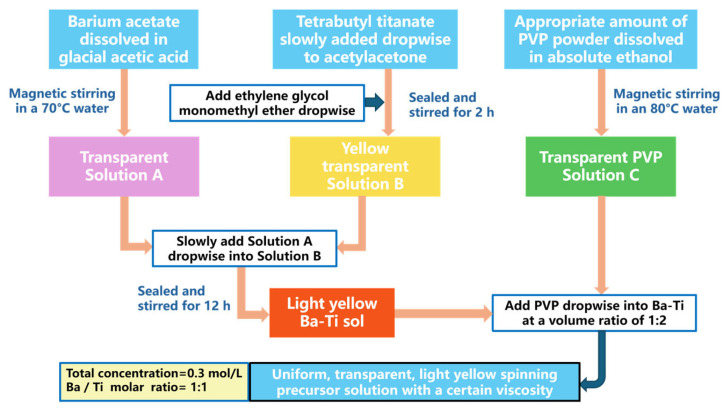
Schematic diagram of the barium titanate sol preparation process.

Solution A (4.644 g barium acetate in 31.84 mL glacial acetic acid) and Solution B (6.12 mL tetrabutyl titanate, 6.12 mL acetylacetone, and 15.92 mL ethylene glycol monomethyl ether) were prepared separately under stirring. Solution A was then added dropwise into Solution B to form a Ba-Ti sol. Subsequently, an ethanolic PVP solution was mixed with the sol at a 1:2 volume ratio. The final electrospinning precursor maintained a 0.3 mol/L total ion concentration and a strict 1:1 Ba/Ti molar ratio. The corresponding barium titanate sol preparation process is illustrated in Figure 1.

#### 2.2.2. Electrospinning

The freshly prepared precursor solution was loaded into a syringe equipped with a 21G needle (inner diameter of 0.51 mm), and the flow rate of the syringe pump was set to 0.5 mL/h. A DC high voltage of 18 kV was applied between the needle and the rotating drum collector, with the tip-to-collector distance set to 15 cm and the drum rotation speed at 1500 rpm. Continuous electrospinning was performed for 8 h under conditions of an ambient temperature of 25 ± 2 °C and a relative humidity below 30% to collect the PVP/BTO composite fiber membrane.

#### 2.2.3. Two-Step Annealing

**Figure 2:**
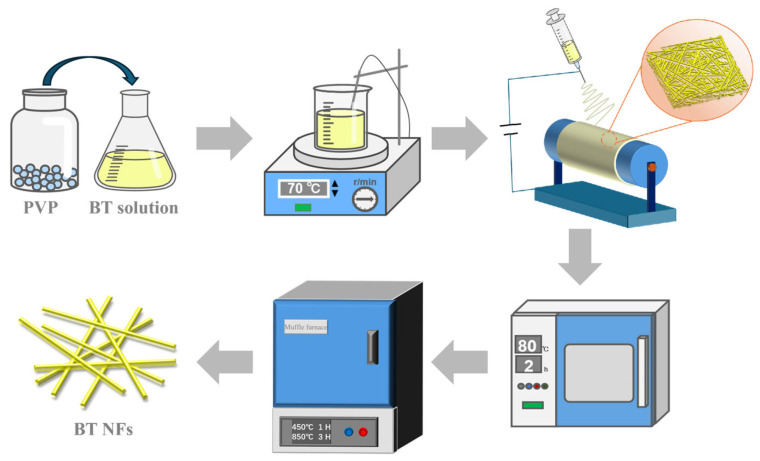
Flowchart of the electrospinning and two-step annealing process.

After being vacuum-dried at 80 °C for 2 h, the collected composite fiber membrane was placed in a muffle furnace for a two-step gradient annealing process. In the first step, it was slowly heated to 450 °C at a heating rate of 2 °C/min and held for 1 h to facilitate the slow oxidative decomposition of PVP and organic matters. In the second step, the temperature was raised to a specific temperature (preferably 850 °C) at a rate of 5 °C/min and maintained for 3 h to promote BTO grain growth and crystalline phase perfection. Finally, one-dimensional BTO nanofibers were obtained after cooling naturally inside the furnace. The preparation process of Electrospinning and annealing is illustrated in Figure 2.

### 2.3. Preparation of PPy@PBT NFs

**Figure 3:**
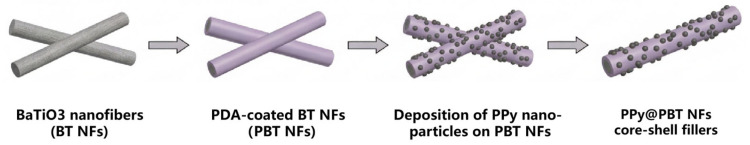
Schematic illustration for the preparation of the integrated core–shell PPy@PBT NFs composite filler.

The synthesis of PPy@PBT NFs consists of two sequential steps: the in situ polymerization of polydopamine, followed by the liquid-phase deposition of polypyrrole. The preparation process is illustrated in Figure 3.

#### 2.3.1. In Situ Coating of Polydopamine

Dopamine hydrochloride (0.4482 g) was dissolved in 100 mL of Tris-HCl buffer (pH = 8.5) to form a 0.05 mol/L solution. Following the addition of 1 g of BTO NFs, the suspension was magnetically stirred in an oil bath at 60 °C for 12 h to facilitate self-polymerization. The PDA@BTO NFs were finally obtained after water washing, centrifugation, and vacuum drying at 80 °C for 24 h.

#### 2.3.2. Liquid-Phase Deposition of Polypyrrole

A total of 0.2 g of FeCl_3_·6H_2_O was dissolved in 5 mL of anhydrous ethanol, into which 0.05 g of PDA@BTO NFs was added. After stirring for 30 min and standing, 2 mL of pyrrole monomer was introduced into the supernatant, followed by continuous stirring for 20 h. The final core–shell structured filler with PPy particles uniformly deposited on the PDA layer (PPy@PBT NFs) was obtained by centrifugation and vacuum drying at 60 °C for 24 h.

### 2.4. Preparation of PPy@PBT NFs/PVDF PEHs

**Figure 4:**
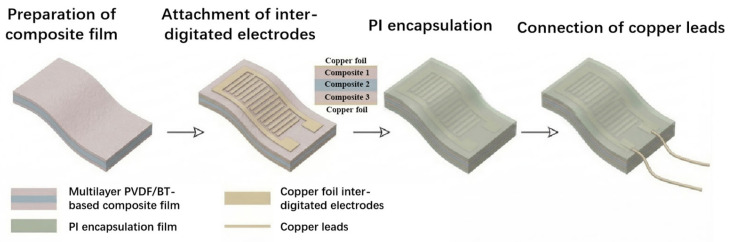
Schematic diagram of the assembly process for the PPy@PBT NFs/PVDF PEHs.

PVDF (0.8 g) was dissolved in a DMF/acetone mixture (7:3 *v*/*v*). Composite solutions containing 2, 4, 6, and 8 wt% PPy@PBT NFs were prepared by 2 h of ultrasonication and 2 h of magnetic stirring at 70 °C. To ensure interfacial physical entanglement, a sandwich-structured membrane was fabricated via layer-by-layer electrospinning of the pure PVDF, composite, and pure PVDF solutions. The spinning parameters included an 18 kV applied voltage, a 15 cm collection distance, a 1.0 mL/h flow rate, and a 2000 rpm drum speed. The as-spun trilayer membranes (~75 μm) were vacuum-dried (80 °C, 2 h) and hot-pressed (120 °C, 8 MPa, 1 h) to obtain dense ~50 μm films (denoted as 2PNF to 8PNF). Finally, flexible piezoelectric energy harvesters (PEHs) were assembled using polyimide (PI) and interdigital copper electrodes (100 μm width, 15 pairs), followed by copper wire attachment and device encapsulation. The assembled process is illustrated in Figure 4.

### 2.5. Characterization

The crystalline structure and phase composition of the synthesized barium titanate nanofibers (BTO NFs) were analyzed using an X-ray diffractometer (XRD, D8 Advance, Bruker, Karlsruhe, Germany) with Cu-Kα radiation (λ = 0.15406 nm) (2θ of 10–80° in steps of 5°/min). Local lattice symmetry and the tetragonal phase were confirmed using a confocal Raman spectrometer (LabRAM Odyssey, HORIBA, Kyoto, Japan). The surface morphology and microstructural evolution of the nanofibers were observed using a field-emission scanning electron microscope (FE-SEM, SU8010, Hitachi, Tokyo, Japan). A high-resolution transmission electron microscope (HRTEM, JEM-2100F, JEOL, Tokyo, Japan) equipped with an energy-dispersive X-ray spectrometer (EDS) was used to characterize the internal continuous structure, core–shell configuration, and elemental mapping of the PDA/PPy-modified BTO NFs. We validated the surface chemical composition and the successful polydopamine (PDA) coating with an X-ray photoelectron spectrometer (XPS, AXIS SUPRA+, Kratos, Stretford, UK). The piezoelectricity of composite fiber films was measured using a quasistatic tester (ZJ-4AN, Institute of Acoustics, Chinese Academy of Sciences, Beijing, China). A heat press (YLJ-100E, Hefei Kejing Material Technology Co., Hefei, China) was used to press the composite fiber film to increase the self-support and density of the composite fiber film. We evaluated PEH output performance with an electrometer (Keithley 6514, Keithley Instruments, Cleveland, OH, USA).

## 3. Results and Discussion

### 3.1. Phase and Structural Evolution of BTO NFs

**Figure 5:**
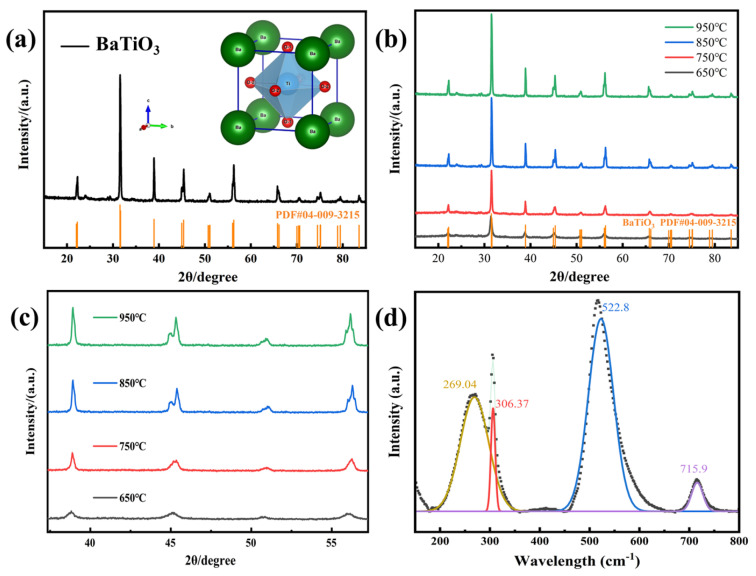
(**a**) XRD patterns and crystal structure schematic of BTO NFs, (**b**) XRD patterns of BTO NFs at different calcination temperatures with (**c**) the corresponding magnified view of the local region, and (**d**) Raman spectra of BTO NFs and the corresponding peak-fitting curves.

The crystal structure of the synthesized barium titanate nanofibers (BTO NFs) was systematically characterized using X-ray diffraction (XRD) and Raman spectroscopy. As shown in Figure 5a, the XRD pattern of the sample calcined at 850 °C exhibits sharp diffraction peaks perfectly matching the standard card for tetragonal perovskite BaTiO_3_ (PDF#04-009-3215). No impurity phases such as BaCO_3_ or TiO_2_ were detected, confirming the complete decomposition of the organic templates and the formation of a highly pure crystalline phase.

The dynamic effect of calcination temperatures (650–950 °C) on the phase evolution is illustrated in Figure 5b,c. At 650 °C, the diffraction peaks are broad and symmetric, displaying typical pseudo-cubic characteristics. As the temperature increases, the crystallites grow significantly, leading to enhanced crystallinity. When the temperature reaches 850 °C and above, the characteristic single peak at 2θ ≈ 45° distinctly splits into a (002) and (200) doublet. This pronounced peak splitting directly evinces the elongation of the c-axis and the contraction of the a-axis, signifying the complete transition of the material from a centrosymmetric pseudo-cubic phase to a non-centrosymmetric tetragonal phase with intrinsic piezoelectric activity. To rule out potential interference from XRD peak broadening at the nanoscale, Raman spectroscopy—which is highly sensitive to local lattice distortions—was employed for further verification (Figure 5d). The deconvoluted spectrum reveals distinct characteristic scattering peaks at 269.04, 306.37, 522.8, and 715.9 cm^−1^. In particular, the sharp “fingerprint” peak at 306.37 cm^−1^ directly reflects the internal asymmetry of the TiO_6_ octahedra. The mutual corroboration between the XRD and Raman results fully demonstrates the excellent tetragonal phase purity of the prepared 1D BTO NFs, laying a solid microstructural foundation for the piezoelectric response of the subsequent flexible composite films.

Based on the (002) and (200) diffraction peaks around 45°, the calculated lattice parameters are c = 4.0293 Å and a = 3.9938 Å, yielding a tetragonality (c/a) = 1.009. Due to the inherent nanoscale size effect—driven by massive specific surface area and internal micro-stress—the lattice undergoes slight distortion, resulting in minor c-axis compression and a-axis expansion. Nevertheless, the BTO NFs maintain exceptionally high tetragonal purity.

The average microcrystalline size is calculated by the Scherrer equation:

(1) D=K λ/β cos θ

where D denotes the microcrystalline size, K represents the shape factor, λ is the incident X-ray wavelength, β is the half-width at half maximum of the diffraction peak, and θ is the Bragg diffraction angle.

**Figure 6:**
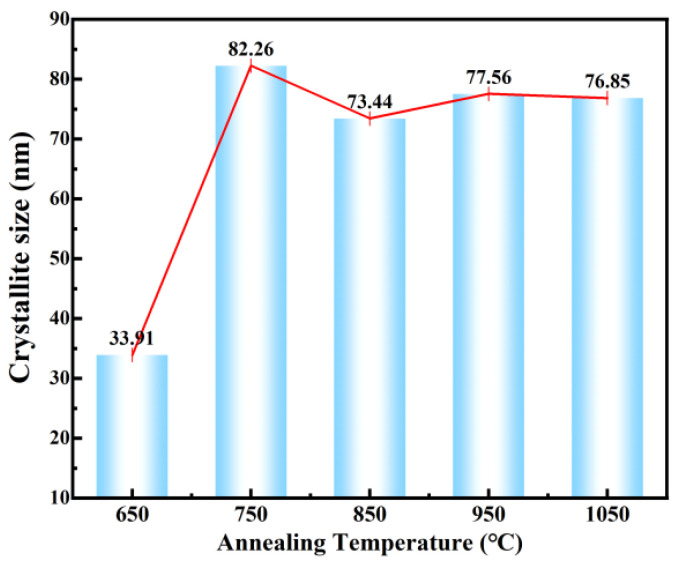
Crystallite size of 1D BTO at different annealing temperatures.

According to the Scherrer equation, the crystallite size increases sharply with calcination temperature, stabilizing at ~77 nm above 750 °C (Figure 6). The slight apparent decline at elevated temperatures is not physical shrinkage, but rather results from peak splitting and broadening induced by the complete pseudo-cubic to tetragonal phase transition. Comparing this crystallite size (~77 nm) with the ~190 nm fiber diameter observed via next SEM indicates that these 1D nanofibers are dense polycrystalline structures assembled from multiple primary crystallites.

### 3.2. Morphological Optimization and Elemental Analysis

**Figure 7:**
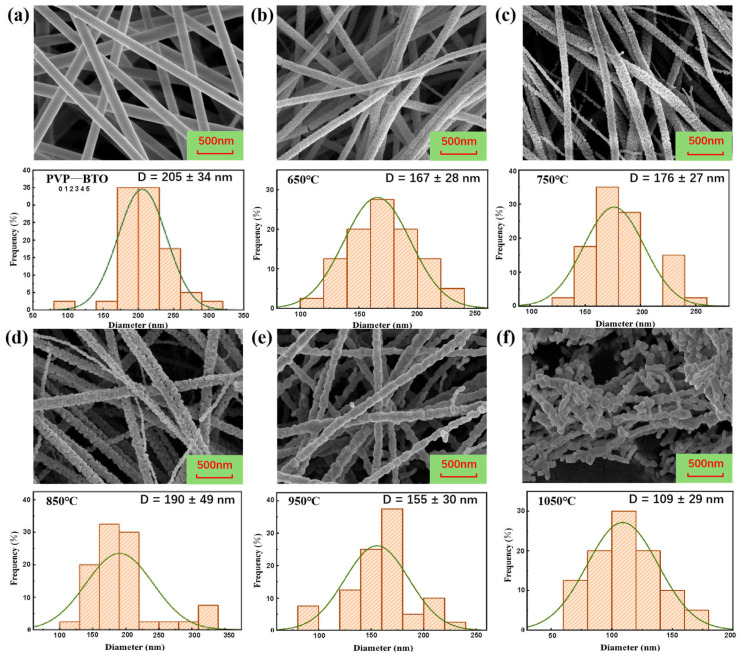
SEM images and corresponding diameter distribution histograms of (**a**) PVP/BTO precursor nanofibers and (**b**–**f**) BTO nanofibers calcined at different temperatures.

Following the confirmation of the optimal tetragonal phase purity at 850 °C, the morphological evolution and elemental composition of the nanofibers were systematically investigated. Scanning electron microscopy (SEM) reveals that the smooth PVP-BTO precursor fibers (average diameter 205 nm) undergo significant structural transformations upon calcination (Figure 7a). At lower temperatures (650–750 °C), polymer decomposition and initial nucleation induce distinct radial shrinkage (Figure 7b,c). At the optimal 850 °C, the BTO NFs develop a robust, continuous 1D “necklace-like” architecture assembled by primary nanocrystals, reaching an average diameter of 190 nm due to grain growth and Ostwald ripening (Figure 7d). Conversely, excessive calcination at 1050 °C triggers Rayleigh instability to minimize surface energy (Figure 7f), resulting in catastrophic fiber fracture and morphological collapse into short, agglomerated clusters (109 nm).

**Figure 8:**
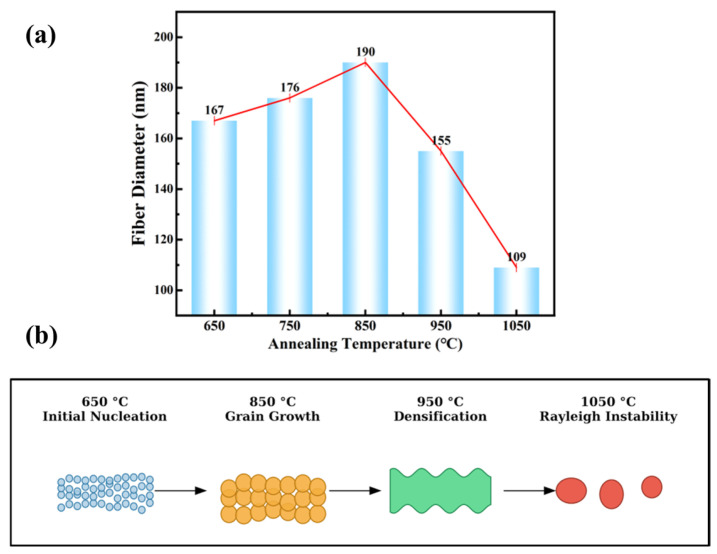
(**a**) Diameters of 1D BTO nanofibers at different annealing temperatures and (**b**) schematic illustration of the diameter variation mechanism.

To elucidate the influence of thermal treatment on the macroscopic dimensions and microstructural stability of the 1D BTO nanofibers, the relationship between annealing temperature and fiber diameter was systematically analyzed. As depicted in Figure 8a, the average diameter of the nanofibers exhibits a distinct non-monotonic trend—initially increasing from 167 nm (at 650 °C) to a peak of 190 nm (at 850 °C), before undergoing a sharp decline to 109 nm at 1050 °C.

This complex morphological evolution is governed by the competition between thermodynamic grain growth, sintering densification, and surface energy minimization. As schematically illustrated in Figure 8b, the morphological evolution of BTO nanofibers is governed by different dominant thermodynamic mechanisms across different temperature ranges.

Between 650 °C and 850 °C, Ostwald ripening becomes the dominant process. At this stage, the average particle size increases and the number of particles decreases, but the particles remain separate from one another; radial grain growth within the 1D physical constraints expands the fiber diameter to a peak of 190 nm, yielding a rough, “necklace-like” structure.

From 850 °C to 950 °C, high-temperature sintering densification dominates. At high temperatures, grain boundaries form between adjacent particles. Through mass transfer processes such as atomic diffusion and grain boundary migration, the particles bond together, causing the pores to shrink and eventually disappear, ultimately forming a dense polycrystal. At this point, the diameter of the dense fibers decreases to 155 nm, but they still retain their one-dimensional continuity.

However, at an extreme 1050 °C, in this case, Rayleigh instability plays a dominant role in the sintering process. The high surface energy of the 1D morphology triggers Rayleigh instability, which leads to localized necking, spheroidization, and the catastrophic fragmentation of the continuous fibers into isolated particulate clusters (109 nm).

Consequently, 850 °C is identified as the optimal annealing temperature, as it perfectly balances sufficient crystallite development with the preservation of robust, continuous 1D structural integrity.

**Figure 9:**
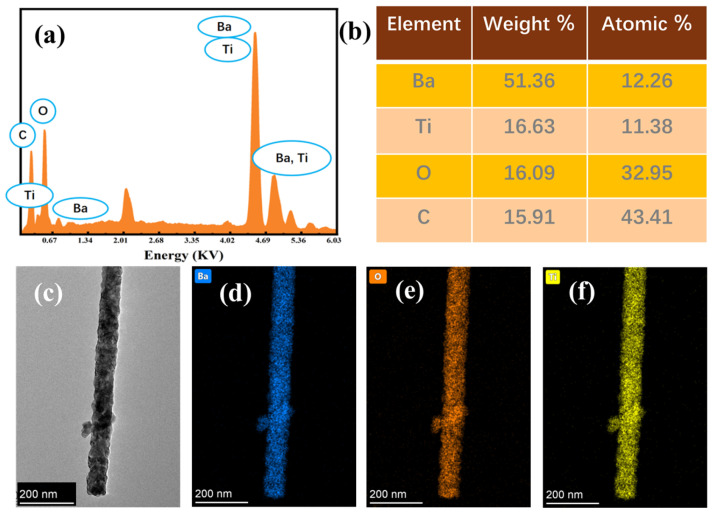
(**a**) EDS spectrum and (**b**) elemental quantitative analysis table of 1D BTO NFs calcined at 850 °C; (**c**) TEM bright-field image of 1D BTO NFs calcined at 850 °C and the corresponding EDS elemental mapping of (**d**) Ba, (**e**) O, and (**f**) Ti elements.

As shown in Figure 9a,b, Energy-dispersive X-ray spectroscopy (EDS) analysis confirms the constituent elements, yielding an atomic Ba/Ti ratio of 1.08. This closely aligns with the ideal 1:1 stoichiometry of perovskite BTO, with the slight deviation attributed to minor surface barium segregation typical during high-temperature annealing. Focusing on the optimal 850 °C sample, transmission electron microscopy (TEM) elucidates a dense, highly continuous polycrystalline 1D skeleton without structural voids (Figure 9c). Furthermore, corresponding EDS elemental mappings demonstrate a highly homogeneous spatial distribution of Ba, Ti, and O along both the radial and longitudinal axes of the nanofiber (Figure 9d–f), strictly precluding any localized elemental segregation.

In summary, the combined SEM, TEM, and EDS analyses verify the successful synthesis of structurally intact, compositionally uniform, and highly continuous 1D BTO NFs at 850 °C. This optimal 1D geometry perfectly complements their high tetragonal crystallinity, providing an ideal microstructural foundation for constructing efficient stress-transfer networks in subsequent piezoelectric composites.

### 3.3. Construction and Mechanism of the Core-Shell PPy@PBT NFs

To overcome the inherent interfacial incompatibility between inorganic ferroelectric fillers and polymer matrices, a bio-inspired, two-step surface modification strategy was employed to construct one-dimensional core–shell PPy@PDA@BTO NFs (denoted as PPy@PBT NFs).

**Figure 10:**
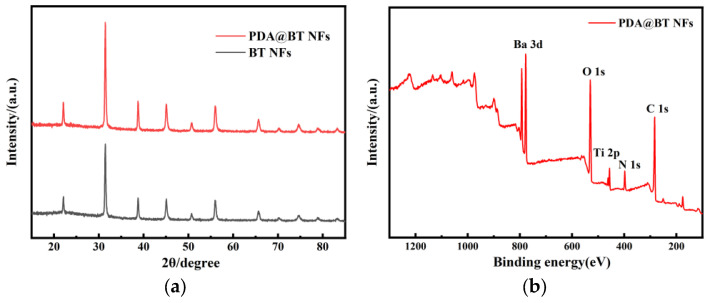
Characterization of the structure and composition of nanofibers before and after surface modification: (**a**) XRD pattern comparison of pure and modified BTO NFs; (**b**) XPS survey spectrum of PDA@BTO NFs.

The successful conformal coating of the polydopamine (PDA) buffer layer was first verified. As shown in Figure 10a, the XRD pattern of the PDA@BT NFs perfectly overlaps with that of pristine BTO NFs, indicating that the mild, weakly alkaline self-polymerization of dopamine completely preserves the non-centrosymmetric tetragonal lattice responsible for piezoelectricity. Since pristine BaTiO_3_ contains no nitrogen, the distinct emergence of the N 1s peak in the XPS survey spectrum (Figure 10b) provides definitive chemical evidence of the PDA coating.

**Figure 11:**
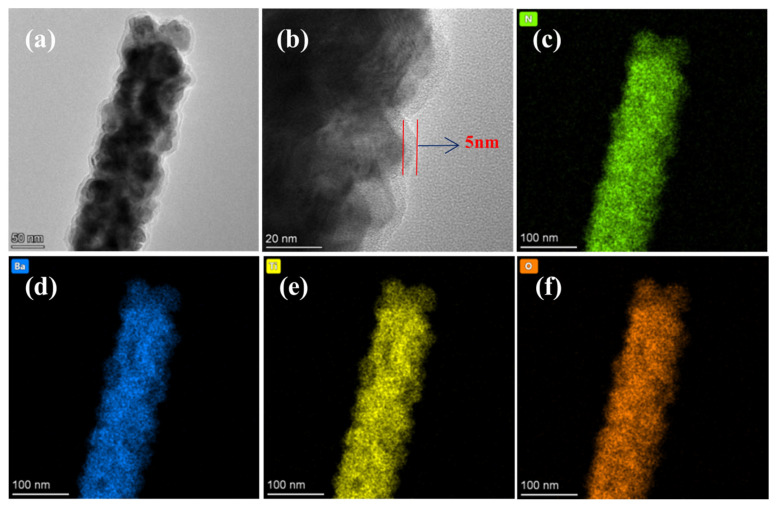
Morphological and compositional characterization of PDA@BT NFs: (**a**) TEM bright-field image; (**b**) high-resolution TEM image showing the 5 nm PDA coating layer; (**c**–**f**) corresponding EDS elemental mappings of N, Ba, Ti, and O elements.

This is intuitively corroborated by the high-resolution TEM observations (Figure 11a). The bright-field TEM image reveals a uniform, amorphous PDA shell with a thickness of 5 nm tightly wrapping the highly crystalline BTO core (Figure 11b). Furthermore, Figure 11c–f provided the corresponding EDS elemental mapping, demonstrating that the N element is distributed with exceptional homogeneity along the entire 1D skeleton, perfectly matching the spatial profiles of Ba, Ti, and O. As shown in Figure 11c, the N signal from the thin PDA layer overlaps with the Ba, Ti, and O signals generated by the internal BTO NFs. This phenomenon is primarily attributed to the fact that the spatial resolution of EDS is limited by the volume of electron beam interaction and the sample thickness. The penetration depth of the electron beam can reach several hundred nanometers, and the excited volume exceeds the 5 nm thick PDA shell layer. Therefore, even if the PDA is strictly confined to the fiber surface, the signal obtained by EDS still represents the volume-averaged information of the entire fiber cross-section.

This proves that PDA undergoes conformal, in situ growth on the BTO surface rather than random precipitation.

**Figure 12:**
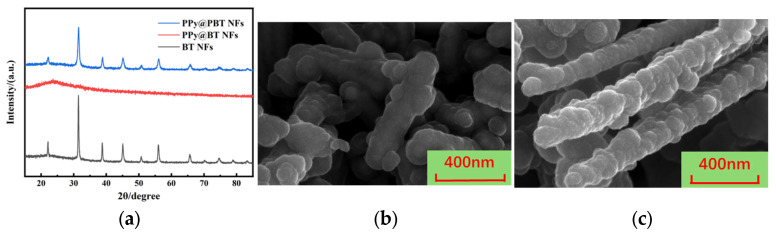
Structural and morphological comparison of BTO nanofibers under different modification strategies: (**a**) XRD patterns; (**b**) SEM image of directly modified PPy@BT NFs; (**c**) SEM image of synergistically modified PPy@PBT NFs.

Crucially, Figure 12 elucidates the indispensable protective mechanism of this PDA buffer layer during the subsequent deposition of the conductive polypyrrole (PPy) shell. When PPy is directly polymerized onto bare BTO NFs (PPy@BT NFs), the characteristic XRD diffraction peaks of the perovskite phase dramatically attenuate and virtually disappear into a broad amorphous halo (Figure 12a).

Morphologically, the corresponding SEM image (Figure 12b) reveals a catastrophic structural collapse, where the continuous 1D fibers are fractured and heavily agglomerated. The underlying mechanism for this destruction is severe acid etching. The in situ polymerization of pyrrole relies on FeCl_3_ as an oxidant, which hydrolyzes to create a highly acidic environment. The pristine perovskite BTO lattice is highly susceptible to proton (H^+^) attack, leading to the rapid dissolution of the inorganic skeleton and the permanent loss of its ferroelectric properties. In stark contrast, the PDA-buffered fibers (PPy@PBT NFs) perfectly retain the sharp, intense XRD peaks of tetragonal BTO (Figure 12a). As evidenced by the SEM image in Figure 12c, the 1D physical morphology remains remarkably intact, with nodular PPy conductive particles uniformly anchoring onto the fiber surface. In this synergistic architecture, the dense, cross-linked PDA shell effectively acts as a diffusion barrier, shielding the vulnerable BTO core from H^+^ penetration during the acidic PPy polymerization. Simultaneously, the abundant catechol and amine functional groups on the PDA surface serve as highly active secondary nucleation sites, promoting the uniform deposition of PPy. Ultimately, this rational core–shell design seamlessly integrates the intrinsic piezoelectricity of the shielded BTO core with the interfacial conductivity of the PPy shell.

### 3.4. Performance Enhancement and Application Potential of PPy@PBT NFs/PVDF PEHs

Building upon the successful synthesis of the 1D core–shell PPy@PBT NFs, their application as multifunctional fillers within a PVDF matrix was investigated to fabricate flexible piezoelectric energy harvesters (PEHs). The incorporation of these hierarchical core–shell fillers dramatically enhances the electromechanical properties of the resulting composite films. Notably, doping the PVDF matrix with an optimal concentration of 4 wt% of the PPy@PBT NFs significantly improves both the intrinsic piezoelectric properties and the macroscopic electrical output performance of the films.

**Figure 13:**
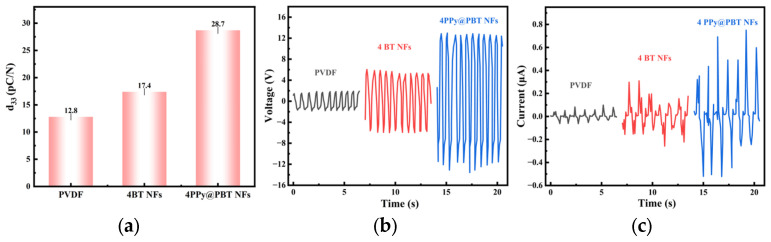
Piezoelectric and electrical output performance of the fabricated PEHs: (**a**) piezoelectric coefficient (d_33_), (**b**) output voltage, and (**c**) output current of pure PVDF, 4 wt% BTO NFs/PVDF, and 4 wt% PPy@PBT NFs/PVDF composite films.

As quantitatively illustrated in Figure 13, the PEH device based on 4 wt% PPy@PBT NFs exhibits a remarkable leap in electromechanical performance. The piezoelectric coefficient d_33_ of the composite films was measured using a quasi-static piezoelectric coefficient tester (ZJ-4AN, Institute of Acoustics, Chinese Academy of Sciences, China). Prior to measurement, all samples were subjected to corona poling at 12 kV/mm and 80 °C for 30 min to align ferroelectric dipoles, followed by 24 h of relaxation at room temperature to eliminate residual surface charges. For each sample, five different positions were tested, and the average value was reported as the final d_33_. As a result, the piezoelectric coefficient (d_33_) of the optimized composite reaches 28.7 pC/N (Figure 13a), which is significantly higher than that of the unmodified 4 wt% BTO NFs composite (17.4 pC/N) and pure PVDF (12.8 pC/N).

At the same time, the output voltage and current were measured using an electrometer (Keithley 6514) under a periodic compressive force generated by a linear motor-driven dynamic testing system. The applied force amplitude was 20 N at a frequency of 2 Hz. A preload of 5 N was applied for 30 s prior to each measurement to eliminate triboelectric contributions and ensure intimate contact between layers.

Correspondingly, the macroscopic electrical outputs under mechanical excitation are drastically amplified. The peak-to-peak output voltage and current of the 4 wt% PPy@PBT NFs device surge to approximately 13 V and 0.7 uA, respectively, vastly outperforming the bare BTO NFs doped counterparts (Figure 13b,c). The slight fluctuation observed in the output current, particularly for the 4 wt% PPy@PBT NFs sample, is attributed to the micro-scale contact resistance variation between the conductive PPy shell and the PVDF matrix under dynamic deformation, as well as localized electric field inhomogeneity caused by minor filler aggregation.

Meanwhile, in the dynamic testing of flexible sensors, non-piezoelectric factors such as triboelectric effects, electrostatic interference in the testing environment, or poor line contact often generate false voltage signals. To strictly prove that the electrical output obtained from the test is purely derived from the intrinsic piezoelectric effect of the 4PNF film, this study conducted a classic polarity reversal (Switching Polarity) test for verification.

**Figure 14:**
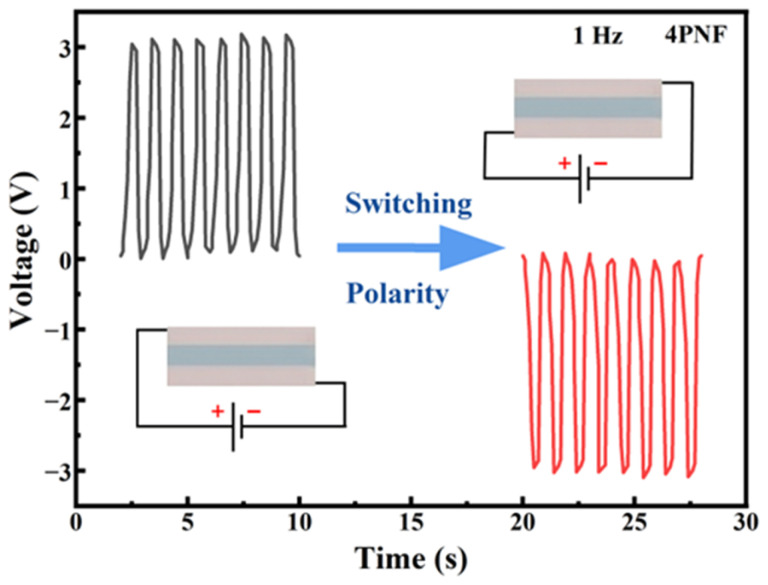
The forward and reverse connections of the sensor output electrical signals.

As illustrated in Figure 14, when the sensor is connected to the oscilloscope in the forward direction, the internal dipole is compressed the moment the device is pressed, inducing a positive charge on the electrode surface; the oscilloscope captures a positive voltage spike. When the pressure is released, the deformation elastically recovers, the charge flows back, and a negative voltage spike is generated. However, when the positive and negative terminals are intentionally swapped—that is, when the sensor is connected to the test terminal in the opposite direction—the output signal waveform undergoes a perfect mirror image reversal. This precise and symmetrical polarity reversal phenomenon completely eliminates interference from non-piezoelectric factors such as triboelectric charging.

**Figure 15:**
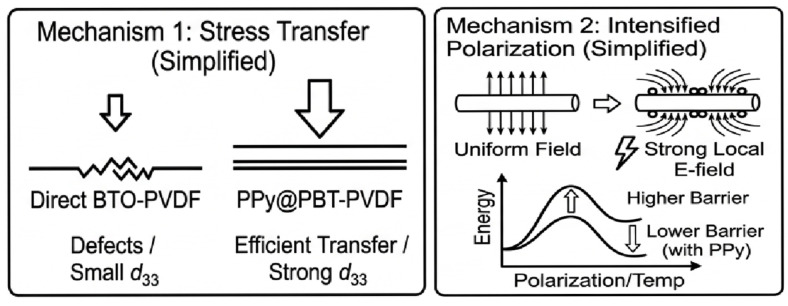
Schematic diagram of the two enhancement mechanisms.

The enhancement mechanisms originate from two synergistic effects (Figure 15):

Firstly, the flexible PDA buffer mitigates the elastic modulus mismatch between the rigid BTO core and the soft PVDF matrix. This minimizes interfacial defects and voids, ensuring that external mechanical stress is optimally transferred to the BTO core to maximize the intrinsic d_33_ response.

Secondly, the conductive PPy nanoparticles function as distributed micro-electrodes within the matrix. Under an applied field, they induce robust Maxwell-Wagner interfacial polarization, which concentrates the local electric field and significantly lowers the energy barrier for ferroelectric dipole alignment in both the BTO and PVDF.

Consequently, the synergistic integration of the robust 1D piezoelectric BTO skeleton, the flexible PDA buffer, and the conductive PPy network culminates in a highly responsive and efficient energy harvesting device. The exceptional performance achieved by the PPy@PBT NFs/PVDF PEHs validates the success of this multi-level in situ coating strategy, laying a solid and promising foundation for the future development and application of high-performance flexible piezoelectric composite materials in self-powered wearable electronics.

In addition, Table 1 summarizes a comparison between this study and recently reported BTO/PVDF piezoelectric systems, including output voltage and filler content. The results indicate that our PPy@PBT/PVDF composite outperforms most previously reported systems in terms of performance, while the lower doping level further ensures the composite’s flexibility, thereby validating the effectiveness of the multi-step surface modification strategy.

## 4. Conclusions

In conclusion, this study successfully overcomes the low polarization efficiency and interfacial incompatibility of traditional inorganic fillers. Highly pure one-dimensional tetragonal BTO nanofibers were synthesized via sol–gel electrospinning and a two-step gradient annealing process. Innovatively, a hierarchical core–shell PPy@PBT NFs composite filler was engineered through the in situ self-polymerization of a polydopamine (PDA) buffer and the subsequent liquid-phase deposition of a conductive polypyrrole (PPy) shell. Doping a PVDF matrix with 4 wt% of these multifunctional fillers dramatically enhanced the device’s electromechanical properties. The optimized flexible piezoelectric energy harvesters achieved a remarkable piezoelectric coefficient of 28.7 pC/N, an output voltage of 13 V, and an output current of 0.7 uA. These leaps in performance are synergistically driven by the PDA layer optimizing interfacial stress transfer and the distributed PPy micro-electrodes intensifying Maxwell-Wagner polarization. Ultimately, this rational multi-level surface modification strategy establishes a robust foundation for developing high-performance flexible piezoelectric composites for next-generation self-powered wearable electronics.

## Figures and Tables

**Table 1:** Comparison with previously reported piezoelectric composite.

Material	Output Voltage	Filler Content	References
Four-layered BTO/PVDF	14.22 V	20 wt%	[25]
PDA@BTO/PVDF	9.30 V	17 wt%	[26]
BTO/PVDF	7.87 V	20 wt%	[27]
BTO NPs/PVDF	7.20 V	10 wt%	[28]
PPy@PBT/PVDF	13.0 V	4 wt%	This work

## Data Availability

The original contributions presented in this study are included in the article. Further inquiries can be directed to the corresponding author.

## References

[1] Fan F.R., Tang W., Wang Z.L. (2016). Flexible nanogenerators for energy harvesting and self-powered electronics. Adv. Mater..

[2] Dagdeviren C., Li Z., Wang Z.L. (2017). Recent progress in flexible and stretchable piezoelectric devices for mechanical energy harvesting, sensing and actuation. Extrem. Mech. Lett..

[3] Acosta M., Novak N., Rojas V., Patel S., Vaish R., Koruza J., Rossetti G.A., Rödel J. (2017). BaTiO_3_-based piezoelectrics: Fundamentals, current status, and perspectives. Appl. Phys. Rev..

[4] Jiang J., Tu S., Fu R., Li J., Hu F., Yan B., Gu Y., Chen S. (2020). Flexible Piezoelectric Pressure Tactile Sensor Based on Electrospun BaTiO_3_. ACS Appl. Mater. Interfaces.

[5] Kubin M., Makreski P., Zanoni M., Gasperini L., Selleri G., Fabiani D., Gualandi C., Bužarovska A. (2023). Effects of nano-sized BaTiO_3_ on microstructural, thermal, mechanical and piezoelectric behavior of electrospun PVDF/BaTiO_3_ nanocomposite mats. Polym. Test..

[6] Shi K.M., Jiang P.K., Sun B., Huang X. (2018). Synergistic effect of graphene nanosheet and BaTiO_3_ nanoparticles on performance enhancement of electrospun PVDF nanofiber mat for flexible piezoelectric nanogenerators. Nano Energy.

[7] Tang H., Lin Y., Song K. (2020). Interfacial stress transfer in BaTiO_3_/PVDF nanocomposites: A finite element study. Compos. Sci. Technol..

[8] Kim H., Lee S., Park K.I. (2019). Silane coupling agent modification of BaTiO_3_ nanoparticles for high-performance piezoelectric nanocomposites. ACS Appl. Mater. Interfaces.

[9] Zhang Y., Liu Y., Wang Z.L. (2015). One-dimensional nanostructures for flexible electronics. Nano Today.

[10] Gao P.X., Lao C.S., Hughes W.L., Wang Z.L. (2005). Three-dimensional interconnected nanowire networks of ZnO. Chem. Phys. Lett..

[11] Ding Y., Liu Y., Zhang Y. (2017). Hydrothermal synthesis of barium titanate nanostructures: From nanoparticles to nanowires. Cryst. Growth Des..

[12] Li D., Xia Y. (2004). Electrospinning of nanofibers: Reinventing the wheel?. Adv. Mater..

[13] Ramakrishna S., Fujihara K., Teo W.E., Yong T., Ma Z., Ramaseshan R. (2006). Electrospun nanofibers: Solving global issues. Mater. Today.

[14] Anga A.P., Dasgupta Ghosh B. (2025). Electrospun PVP-barium titanate nanofibers: Tailoring morphology and enhancing piezoelectric performance for advanced applications. Ceram. Int..

[15] Yuh J., Nino J.C., Sigmund W.M. (2005). Synthesis of barium titanate (BaTiO_3_) nanofibers via electrospinning. Mater. Lett..

[16] Wang Y., Liu Z., Chen X. (2019). Balancing organic template removal and phase evolution in electrospun ceramic nanofibers. Ceram. Int..

[17] Pan C., Zhang Y., Wang Z.L. (2018). Unraveling the crystallization kinetics of electrospun BaTiO_3_ nanofibers during annealing. J. Mater. Chem. C.

[18] Zhao J.Y., Li Z.L., Lv S.W., Wang M.X., Li C.P., Li X., Chen H.Y., Li M.X., Chen X.C., Wang F.F. (2023). Electrospun advanced nanomaterials for in situ transmission electron microscopy: Progress and perspectives. InfoMat.

[19] He Y., Li J., Zhang H. (2022). Heating rate-dependent microstructure evolution in sol-gel derived BaTiO_3_ nanofibers. J. Am. Ceram. Soc..

[20] Saber N., Araby S., Meng Q.S., Hsu H.Y., Yan C., Azari S., Lee S.H., Xu Y.N., Ma J., Yu S. (2014). Superior piezoelectric composite films: Taking advantage of carbon nanomaterials. Nanotechnology.

[21] Zhang X., Wang L., Chen Y. (2018). Polypyrrole coating on ferroelectric ceramics: Corrosion effects and structural stability. J. Mater. Chem. A.

[22] Lee H., Dellatore S.M., Miller W.M., Messersmith P.B. (2007). Mussel-inspired surface chemistry for multifunctional coatings. Science.

[23] Liu Y., Ai K., Lu L. (2014). Polydopamine and its derivative materials: Synthesis and promising applications in energy, environmental, and biomedical fields. Chem. Rev..

[24] Guan X., Xu B., Gong J. (2020). Hierarchically architected polydopamine modified BaTiO_3_@P(VDF-TrFE) nanocomposite fiber mats for flexible piezoelectric nanogenerators and self-powered sensors. Nano Energy.

[25] Li Y., Su X., Liang K., Luo C., Li P., Hu J., Li G., Jiang H., Wang K. (2021). Multi-layered BTO/PVDF nanogenerator with highly enhanced performance induced by interlaminar electric field. Microelectron. Eng..

[26] Ye Y., Hong P., Xie G., Jiang Y. (2020). Flexible piezoelectric pressure sensor based on polydopamine-modified BaTiO_3_/PVDF composite film for human motion monitoring. Sens. Actuators A-Phys..

[27] Kim H.T., Park S.S. (2023). Effects of Mixing Ratio and Poling on Output Characteristics of BaTiO_3_-Poly Vinylidene Fluoride Composite Piezoelectric Generators. Korean J. Mater. Res..

[28] Kim H.J. (2021). Piezoelectric Nanogenerator Based on Lead-Free Flexible PVDF-Barium Titanate Composite Films for Driving Low Power Electronics. Crystals.

